# The Time Course of the Pupillary Response to Auditory Emotions in Pseudospeech, Music, and Vocalizations

**DOI:** 10.1177/23312165251365824

**Published:** 2025-08-10

**Authors:** Julie Kirwan, Deniz Başkent, Anita Wagner

**Affiliations:** 1 Department of Otorhinolaryngology/Head and Neck Surgery, University Medical Center Groningen, University of Groningen, Groningen, The Netherlands; 2 Research School of Behavioural and Cognitive Neurosciences, Graduate School of Medical Sciences, University of Groningen, Groningen, The Netherlands

**Keywords:** pupillometry, pupil dilation, pseudospeech, valence, growth curve analysis

## Abstract

Emotions can be communicated through visual and dynamic characteristics such as smiles and gestures, but also through auditory channels such as laughter, music, and human speech. Pupil dilation has become a notable marker for visual emotion processing; however the pupil's sensitivity to emotional sounds, specifically speech, remains largely underexplored. This study investigated the processing of emotional pseudospeech, which are speech-like sentences devoid of semantic content. We measured participants’ pupil dilations while they listened to pseudospeech, music, and human vocalizations, and subsequently performed an emotion recognition task. Our results showed that emotional pseudospeech can trigger increases of pupil dilation compared to neutral pseudospeech, supporting the use of pupillometry as a tool for indexing prosodic emotion processing in the absence of semantics. However, pupil responses to pseudospeech were smaller and slower than the responses evoked by human vocalizations. The pupillary response was not sensitive enough to distinguish between emotion categories in pseudospeech, but pupil dilations to music and vocalizations reflected some emotion-specific pupillary curves. The valence of the stimulus had a stronger overall influence on pupil size than arousal. These results highlight the potential for pupillometry in studying auditory emotion processing and provide a foundation for contextualizing pseudospeech alongside other affective auditory stimuli.

## Introduction

Speech is a hugely important means of communication. In order for speech communication to be effective, we need to understand not only the words that an individual is saying, but also the emotion behind the words. There are clinical populations where the recognition of these emotions may be diminished, such as people with hearing impairments or autism (Christensen et al., 2019; Dennis et al., 2000). For these groups, explicit recognition of emotions can be unreliable or even absent, leading to significant barriers in their social and emotional functioning. In this study, we investigate pupil dilation as a physiological marker of auditory emotion processing in pseudospeech, instrumental music, and non-verbal human vocalizations (such as laughter and cries).

The pupillary response has been widely used as an implicit measure of cognitive and emotional processing (Granholm & Steinhauer, 2004; Hess & Polt, 1964; Hoeks & Levelt, 1993). Researchers have extensively investigated this response in visual emotion processing (Bradley et al., 2008; Partala & Surakka, 2003; Snowden et al., 2016), but also for affective auditory sounds like music and vocalizations (Gingras et al., 2015; Oliva & Anikin, 2018). In contrast, emotional speech has received far less attention in pupillometry research, but the few studies on this topic have shown that emotional speech can also evoke an increase in pupil dilation (Jürgens, 2018; Kuchinke et al., 2011). Both of these studies showed that semantically neutral speech elicits a pupillary response in the listener that is greater when presented with emotional prosody compared to neutral prosody. However, quantifying speech emotion processing through pupil dilation is challenging, since speech is a complex signal that conveys more information than emotions alone. In addition, pupil dilation is commonly used to index listening effort and auditory sentence processing in speech (Aydın & Uzun, 2023; Engelhardt et al., 2010; Wagner et al., 2019). If pupil dilation can index emotion processing to prosodic emotional speech in the absence of any semantic content, we can isolate emotion processing from general processing effort.

Given that pupil dilation can index emotional processing in speech, it becomes important to isolate which speech features drive this effect. Speech can contain emotional information in the form of paralinguistics, prosody, and semantics. The combination of these channels is informative, and while listeners naturally integrate this information, there exists evidence for both a prosodic and semantic dominance (Ben-David et al., 2016; Kotz & Paulmann, 2007). To reduce the confound of having conflicting semantic and prosodic emotional information, neuroimaging and behavioral studies employ the use of pseudosentences, consisting of pseudowords (Brosch et al., 2009; Nagels et al., 2020). Another method to separate these two channels is through filtering the speech signal. For instance, Kotz et al. (2003) used a PURR-filter (Prosody Unveiling through Restricted Representation) to remove segmental and lexical content while preserving the acoustic parameters of the signal (Sonntag & Portele, 1998). In this study, we have used non-semantic prosodic pseudospeech, which preserves prosody while eliminating semantic content (Lausen & Hammerschmidt, 2020).

Pupil dilation can reflect autonomic, emotion, semantic, and cognitive processing (Francis & Love, 2020). Pupil dilation can reflect sympathetic activation caused by a highly arousing stimulus, but it can also reflect parasympathetic inhibition (Mcdougal & Gamlin, 2015). It captures both bottom-up processes, such as autonomic arousal, and top-down processes, such as mental effort (Granholm & Steinhauer, 2004). Although these components cannot yet be fully disentangled, we can investigate how pseudospeech relates to two other semantically-free emotional sounds, human vocalizations and instrumental music. By directly comparing emotional responses to prosodic pseudospeech, music, and nonverbal vocalizations, our study provides a cross-domain perspective on auditory emotion processing.

Non-verbal human vocalizations are extremely effective in conveying a speaker's internal state, with conscious processing doing little to improve the accuracy of emotion recognition (Lima et al., 2019). From event-related potential (ERP) research, we see that listeners encode emotional vocalizations as early as 100 ms after stimulus onset, a faster response than emotional speech (Pell et al., 2015; Sauter & Eimer, 2010). As vocalizations are widely associated with eliciting physiological arousal in the listener (Azhari et al., 2018; Groh & Roisman, 2009), we suspect that the pupillary response to human vocalizations is reflecting autonomic arousal. Jürgens et al. (2018) showed that skin conductance, a measure of autonomic arousal, was modulated by affective bursts, but was not modulated by emotional speech. Affective bursts are sounds such as alarms and bombs, and while different from human vocalizations, they clearly induce emotional arousal in the listener.

Emotional music, like speech, evokes a pupillary response in the listener (Gingras et al., 2015) and pupils dilate more for vocal than instrumental music, indicating the higher relevance of vocal information for the listener (Weiss et al., 2016). The complexity of the stimulus also plays a role: in a study investigating the pupillary response to poetic language, it was shown that aesthetic poetry, which is complex and subtle in its emotional meaning, resulted in a slower pupillary response than comical poetry, which is more easily perceptible (Niikuni et al., 2022). We therefore expect a faster or larger pupillary response for more emotionally salient information, such as vocalizations, than for non-vocal music and pseudospeech.

Emotions can be categorized into labels such as happy, sad, or fearful. Alternatively, the Circumplex model of affect describes emotions along continuous valence and arousal dimensions (Posner et al., 2005; Russell, 1980). Pupillometry research has employed both models of emotion, with mixed results as to how pupil size is modulated by valence, arousal, or specific emotion categories (Kret et al., 2013; Oliva & Anikin, 2018; Partala & Surakka, 2003). Some studies suggest pupil dilation is primarily driven by arousal, with larger responses to highly arousing sound stimuli (Partala et al., 2000), while others find that negative valence elicits greater or more sustained pupil dilation (Burley et al., 2017; Geangu et al., 2011). The valence intensity also predicts pupil size, with responses independent of whether the stimulus is positive or negative (Bradley et al., 2008; Oliva & Anikin, 2018), and this pattern extends to semantically neutral speech with emotional prosody (Kuchinke et al., 2011).

In this study, we investigate two research questions.

1. Is emotion processing reflected in the pupillary response to emotional pseudosentences?
2. How does the pupillary response to emotional pseudosentences compare to non-word human vocalizations and instrumental music?

We used Dutch accented pseudosentences (Bänziger et al., 2012; Nagels et al., 2020), human vocalizations from the Montreal Affective Voices (MAV) (Belin et al., 2008), and the Musical Affective Bursts (MEB) (Paquette et al., 2013), which were developed to be a musical version of the MAV stimuli. We collected both pupillometry and behavioral measures of emotion. Our participants were presented with a forced-choice task asking them to identify which emotion was heard in the stimulus, and, in addition, we collected valence and arousal ratings of the stimuli.

Our primary hypotheses were:

1. Emotional pseudosentences will elicit greater pupil dilation than neutral pseudosentences, reflecting the processing of emotion prosody.
2. Human vocalizations will trigger greater and earlier pupil dilation responses, consistent with autonomic arousal.
3. The pupil response will be highly dependent on the type of stimulus, valence, arousal, and emotion category.

## Methods

### Ethics Statement

The experimental procedures were given ethical approval from the Medical Ethical Committee of the University Medical Center Groningen (UMCG). Participants were informed of the study procedures both verbally and through information letters, and written informed consent was collected from each participant prior to the experiment.

### Participants

Twenty-one native Dutch participants were recruited (7 male; mean age 23 years; age range 18–30 years), the majority were students (*n* = 16) and received 8 euro/hour for their participation. Inclusion criteria were: native Dutch speakers, no early second-language acquisition (<3 years), and no reported dyslexia or autism. The participants’ hearing thresholds were tested for normal hearing, at or below 20 dB HL (frequency range 125 Hz–4 kHz) and had normal or corrected-to-normal vision.

### Stimuli

There were three different types of stimuli used in the experiment; pseudospeech, music, and vocalizations. Stimuli were RMS-normalized in overall amplitude across all materials. The sound presentation level was fixed at around 65 dB SPL. Given the varying spectro-temporal properties of signals across different stimulus sets, each stimulus set was separately calibrated with a manikin (KEMAR, GRAS) and a sound-pressure level meter (Brüel & Kjær, Svan 979).

#### Pseudospeech

The pseudospeech stimuli consisted of 48 recordings produced by six speakers (3 male), expressing four emotions (anger, happiness, sadness, neutral). The sentences were taken from validated pseudo-linguistic materials (Bänziger et al., 2012) and recordings were made in a sound-attenuating room at 44.1 kHz. The stimuli were informally validated in an online forced-choice task (Nagels et al., 2020). Stimulus duration ranged from 1159 to 2147 ms. The mean duration (± standard error) was 1419 ± 47 ms for anger, 1546 ± 40 ms for happiness, 1580 ± 72 ms for neutral, and 1585 ± 68 ms for sadness.

#### Music

The music stimuli consisted of 2 instruments (violin and clarinet), 6 pieces, and 4 emotions (fear, happiness, sadness, neutral), totaling 48 items. The music stimuli were selected from the MEB (Paquette et al., 2013), which were produced with the intention of being a musical version of the MAV (Belin et al., 2008). Paquette et al. (2013) validated the database with 60 participants (19 males) in an online validation. Stimulus duration ranged from 400 to 3110 ms. The mean duration (± standard error) was 928 ± 59 ms for fear, 1529 ± 95 ms for happiness, 1421 ± 70 ms for neutral, and 2608 ± 136 ms for sadness.

#### Vocalizations

The vocalization stimuli consisted of 10 speakers (5 male) producing the utterance *ah* (/**ɑ**/) in 6 emotions (anger, fear, happiness, sadness, pleasure, neutral), totaling 60 items. The vocalizations used in the experiment were a subset of the MAV and were formally validated (Belin et al., 2008). Stimulus duration ranged from 240 to 3600 ms. The mean duration (± standard error) was 851 ± 109 ms for anger, 580 ± 59 ms for fear, 1402 ± 175 ms for happiness, 932 ± 141 ms for neutral, 1363 ± 142 ms for pleasure, and 2135 ± 236 ms for sadness.

### Procedure

Prior to the experiment, participants were given instructions on how to make their judgements of valence and arousal for the stimuli. The term valence was described to them as the degree of positive or negative emotion conveyed by the sound, and arousal as how much excitation is elicited by the emotion in the sound. The participants were given examples of the extremes of the scales from each stimulus set, and were informed that they would choose the emotion category (happy, sad etc.) that best represented the emotion in the sound. However, participants were not given descriptions of the meaning of the emotion categories.

Participants were seated in a sound-treated, dimly-lit room where the lighting was kept constant throughout the experiment. A head-mounted Eyelink II (SR Research) eye-tracker was used for all participants. The procedure started with a short practice block (20 trials, all stimulus types), to familiarize participants with the rating procedure and the range of stimulus types. The experimenter was present during this period and participants were allowed to ask questions about the experiment and the rating scales. Following the practice block, the three experimental blocks (pseudospeech, music, vocalizations) were presented in a random order.

For each trial, a fixation cross was presented 1 s prior to the stimulus onset, and it remained until 3 s after the stimulus offset. Participants were instructed to minimize blinking and focus on the cross while it was presented. After stimulus presentation, participants rated valence on a continuous scale (1 = very negative; 9 = very positive), and arousal (1 = low arousal; 9 = high arousal). Finally, the participant performed a forced-choice task (pseudospeech and music: 4 categories, vocalizations: 6 categories) where they chose the word that best represented the emotion conveyed in the sound. Participants used a mouse to make their selection, and before the beginning of the next trial, a screen instructing the participants to blink was presented.

### Pupil Data Preprocessing

Matlab (version R2017a) was used to carry out the preprocessing of the pupil data. Firstly, each participant's raw pupil data was divided into trials, which started from 1 s before the onset to three seconds after the onset of the stimulus. Then, each trial was checked for blinks exceeding 300 ms. Trials with blinks longer than 300 ms were discarded (4.5% of all data) to avoid artefacts from interpolation.

Dilation outliers were detected using the median absolute deviation, which is a robust metric to detect blinks in pupillometry data (Kret & Sjak-Shie, 2019; Leys et al., 2013). Samples exceeding a velocity threshold of 16 (in line with pupil dilation limits; Mathôt et al., 2018) were discarded. The missing values were interpolated via a piecewise cubic hermite interpolating polynomial. The pre-trial baseline was obtained by taking the average of the pupil size in the 1 s prior to the stimulus onset. The event-related pupil dilation (ERPD) was calculated from the following equation (Wagner et al., 2019):

$$ERPD=\frac{\;pupil\;size-baseline}{baseline\;}\times 100$$

This ERPD provides the percentage change in pupil dilation relative to the pre-trial baseline. Next, we smoothed our data by reducing the frequency from 250 to 50 Hz through averaging consecutive data points into bins of 20 ms (effectively over 5 data points).

## Results

### Behavioral Data

The behavioral data were collected to confirm the categorical and dimensional models of emotions in our three stimulus types: pseudospeech, music, and vocalizations. The music (MEB) and vocalization (MAV) stimulus sets were previously validated for both dimensional and categorical models of emotion, while the pseudospeech (EmoHI) stimuli were validated only for the categorical model of emotion (Belin et al., 2008; Nagels et al., 2020; Paquette et al., 2013). Since these three validations were done separately, we collected both dimensional and categorical ratings of emotion to allow direct, cross-modal comparisons. The statistical analyses and plotting were performed in Rstudio (www.R-project.org, R version 4.2.2). Linear mixed effects models (LMER) and generalized linear mixed effects models (GLMER) were fitted using the lmer and glmer function of the lme4 package respectively (version 1.1–35.1) (Bates et al., 2015). We tested if each term in our models improved the model fit via likelihood ratio tests until we ended up with the most parsimonious model, this included testing for random intercepts and random slopes, up until the most complex random effects structure. The lmerTest package was used to calculate *p*-values based on Satterthwaite's method (Kuznetsova et al., 2017). We tested the LMER model assumptions for (1) normality of residuals, (2) linearity (for models with continuous predictors), and (3) homogeneity of variances via diagnostic plots. Weights were added to some LMER models to stabilize variance.

#### Categorical Ratings

**Figure 1.**
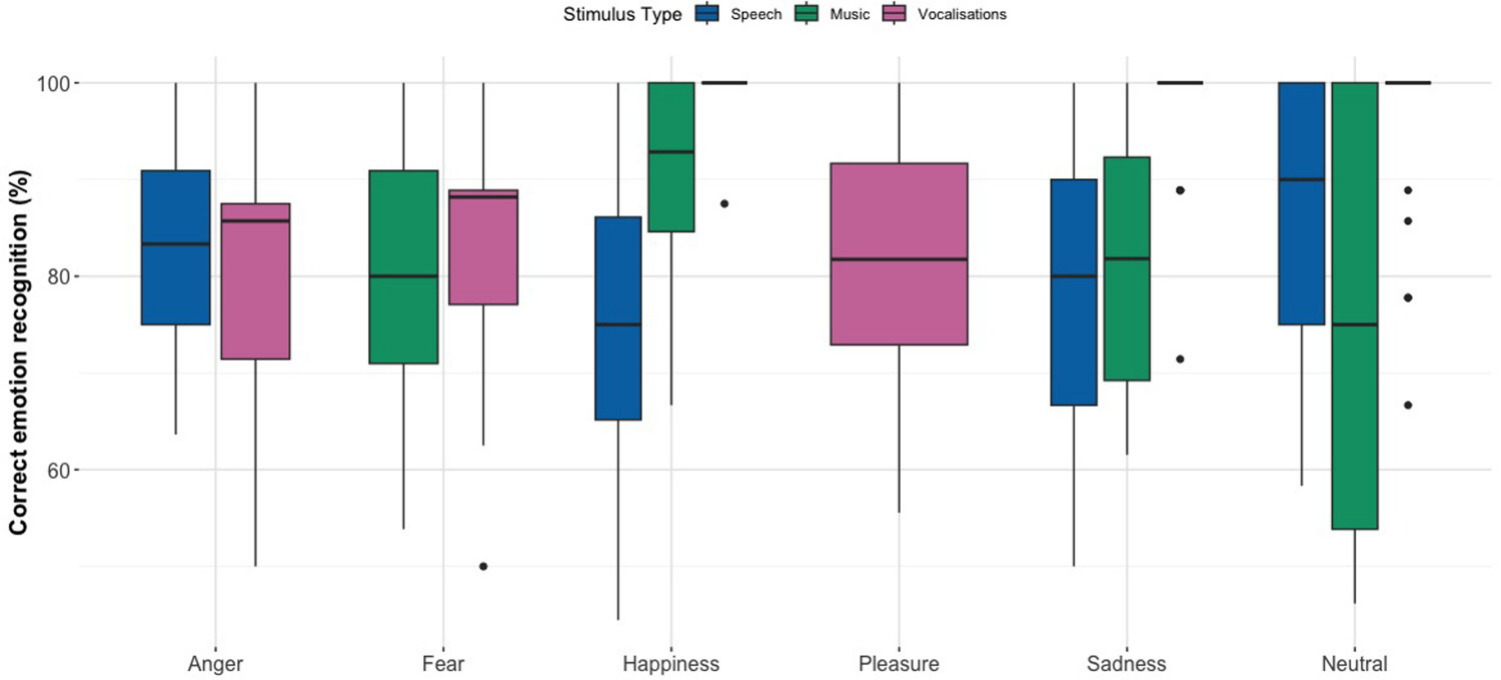
Box Plots Displaying Percent Correct Emotion Recognition Scores for Pseudospeech, Music, and Vocalizations. Horizontal Lines in Bold Indicate Medians, Boxes Indicate Data Within the 25th to 75th Percentile, Whiskers Indicate Data Within the 5th to 95th Percentile, and Circles Indicate Outliers.

Figure 1 shows the percent correct recognition scores for each emotion category across stimulus types. In general, participants correctly identified emotion categories above chance level. A notable confusion was between happiness and pleasure in vocalizations (21% and 13% accuracy, respectively), likely due to their shared positive valence and the fact that no explicit instructions for emotion labels were given to the participants. In order to reduce the statistical error, we grouped together the happiness and pleasure categories, meaning that if a participant rated happiness as pleasure, or vice versa, we concluded this as being a correct answer. Confusion matrices can be found in the Supplemental material (Supplemental Figure 1 ‘Speech’; Supplemental Figure 2 ‘Music’; and Supplemental Figure 3 ‘Vocalizations’).

For the pseudospeech stimuli, participants’ scored above the chance level of 25% for all four categories, namely, neutral (86.9%, SE = 0.022), sadness (78.3%, SE = 0.027), anger (83.3%, SE = 0.025), and happiness (75.6%, SE = 0.030). For the music stimuli, participants’ scored well above the chance level of 25% for all four categories, namely, neutral (76.8%, SE = 0.028), sadness (81.0%, SE = 0.024), fear (78.0%, SE = 0.028), and happiness (91.0%, SE = 0.017). The vocalization ratings show high accuracy scores above chance level (16.7%) for the six categories; neutral (95.0%, SE = 0.016), sadness (96.6%, SE = 0.014), anger (80.3%, SE = 0.032), fear (82.6%, SE = 0.029), happiness (99.4%, SE = 0.006), and pleasure (81.2%, SE = 0.034).

We removed extreme outliers based on the z-score method over four iterations, where a z-score of less than −3 or greater than 3 for a given data point was removed, resulting in two audio files being removed from the dataset (overall recognition rates of 0% and 10% across all participants) along with several participant-emotion combinations. A GLMER revealed a significant interaction between emotion category and stimulus type on recognition accuracy, χ²(6) = 27.253, *p* < .001. Notably, neutral emotions were more difficult to recognize in music (Est = −1.093, SE = 0.528, *p* = .0386), while sadness was most easily recognized in vocalizations (Est = –2.141, SE = 0.732, *p* = .00347). Full model estimates and additional comparisons are provided in Supplemental Tables 1(a) to (e), and the random effect variance estimate is provided in Supplemental Table 1(f).

#### Valence and Arousal Ratings

**Figure 2.**
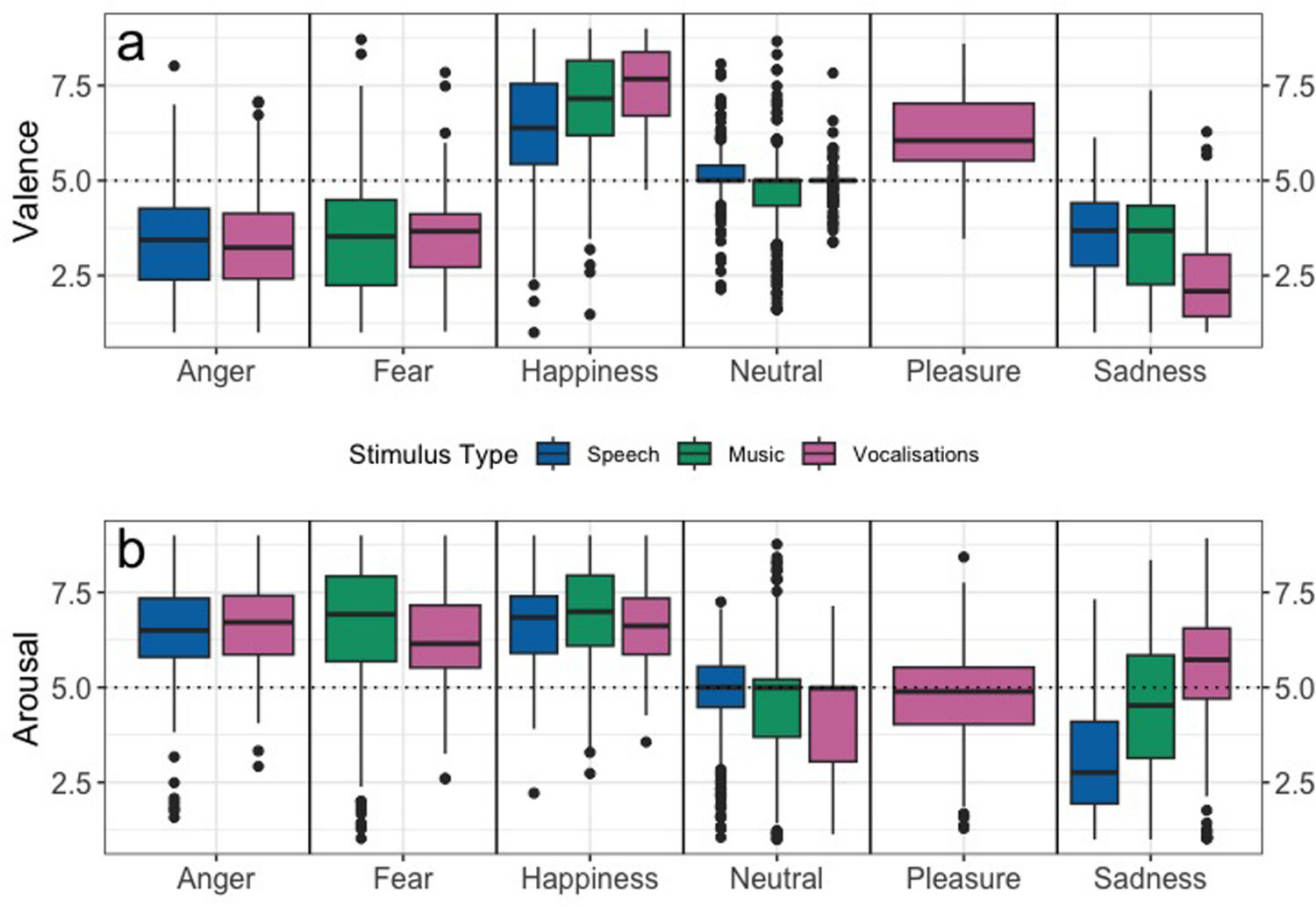
Participant Ratings of (a) Valence (Top Panel) and (b) Arousal (Bottom Panel) for Each Stimulus Type; Pseudospeech, Music, and Vocalizations. Horizontal Lines in Bold Indicate Medians, Boxes Indicate Data Within the 25th to 75th Percentile, Whiskers Indicate Data Within the 5th to 95th Percentile, and Circles Indicate Outliers. Values Range From 1 to 9 and the Dotted Line at 5 Represents the Midway Point.

Figure 2 shows the participants’ ratings of valence and arousal for all three stimuli types of pseudospeech, music, and vocalizations. The valence and arousal data were first analyzed on an ordinal scale, by binning the responses and running the model as an ordinal logistic mixed effect model. But the model assumptions were violated and the data were more suited as a linear model, so the analyses were done with a linear mixed-effects model. A significant interaction between stimulus type and emotion category influenced valence ratings, χ²(6) = 65.203, *p* < .001. Music slightly decreased valence compared to pseudospeech (Est = –0.397, SE = 0.181, *p* = .0295), while both music (Est = 0.949, SE = 0.263, *p* < .001) and vocalizations increased valence when associated with happiness (Est = 1.151, SE = 0.263, *p* < .001). The full model estimates are provided in Supplemental Table 2(a) and the random effect variance estimates in Supplemental Table 2(b).

Similarly, for arousal, an interaction effect of stimulus type and emotion was found to significantly improve model fit compared to a model without the interaction, χ^2^(6) = 71.54, *p* < .001. For stimulus type, vocalizations (Est = –0.448, SE = 0.281, *p* = .0846) and music (Est = −0.116, SE = 0.263, *p* = .6593) did not differ significantly from pseudospeech. There was a strong positive interaction effect of sadness with vocalizations (Est = 2.973, SE = 0.381, *p* < .001) and music (Est = 1.543, SE = 0.340, *p* < .001), revealing that arousal increased significantly. The interaction between anger and vocalizations (Est = 0.835, SE = 0.387, *p* = .0351) was also significant. The full model estimates are provided in Supplemental Table 3(a) and the random effect variance estimates in Supplemental Table 3(b).

### Pupillometry Data

We analyzed ERPD in response to emotional pseudospeech, music, and vocalizations using growth curve analysis (GCA; Mirman, 2014). GCA allows us to capture the time course of pupil dilation, via linear, quadratic, and cubic polynomial time terms. The linear time term represents the overall slope of the pupil data (larger values indicating greater slope), the quadratic term reflects the symmetricity in the peak pupil dilation (negative values indicating a sharper peak) and the cubic term is both a qualifier of the linear term and the extent to which a second inflection point is represented in the tails, where more positive values indicate a ‘sharper’ pupil response (McGarrigle et al., 2017; Neagu et al., 2023; Wagner et al., 2019). The statistical analyses and plotting were performed in Rstudio (www.R-project.org, R version 4.4.2). Pairwise post hoc tests were conducted using the glht function of the multcomp package (Hothorn et al., 2008). We used linear mixed effects regression models from the lme4 package (Bates et al., 2015) and ran different models for the emotion categories and the dimensional model of emotion. We included valence and arousal as centered, continuous variables. Each stimuli set was analyzed using the same time window, which was the shortest trial length (0 to 3240 ms after stimulus onset). Stepwise model comparison in conjunction with visual inspection was used to determine the best terms to use in each model. We started with the null model and subsequently added effects until we found the most parsimonious model. We included maximal random effects structures with random slopes and intercepts but subsequently removed higher order terms due to convergence errors. We looked at the fit of the model to the data so as to avoid overfitting. Statistical significance for the individual parameter estimates was assessed by using the normal approximation (i.e. treating the *t-*value as a *z-*value).

**Figure 3.**
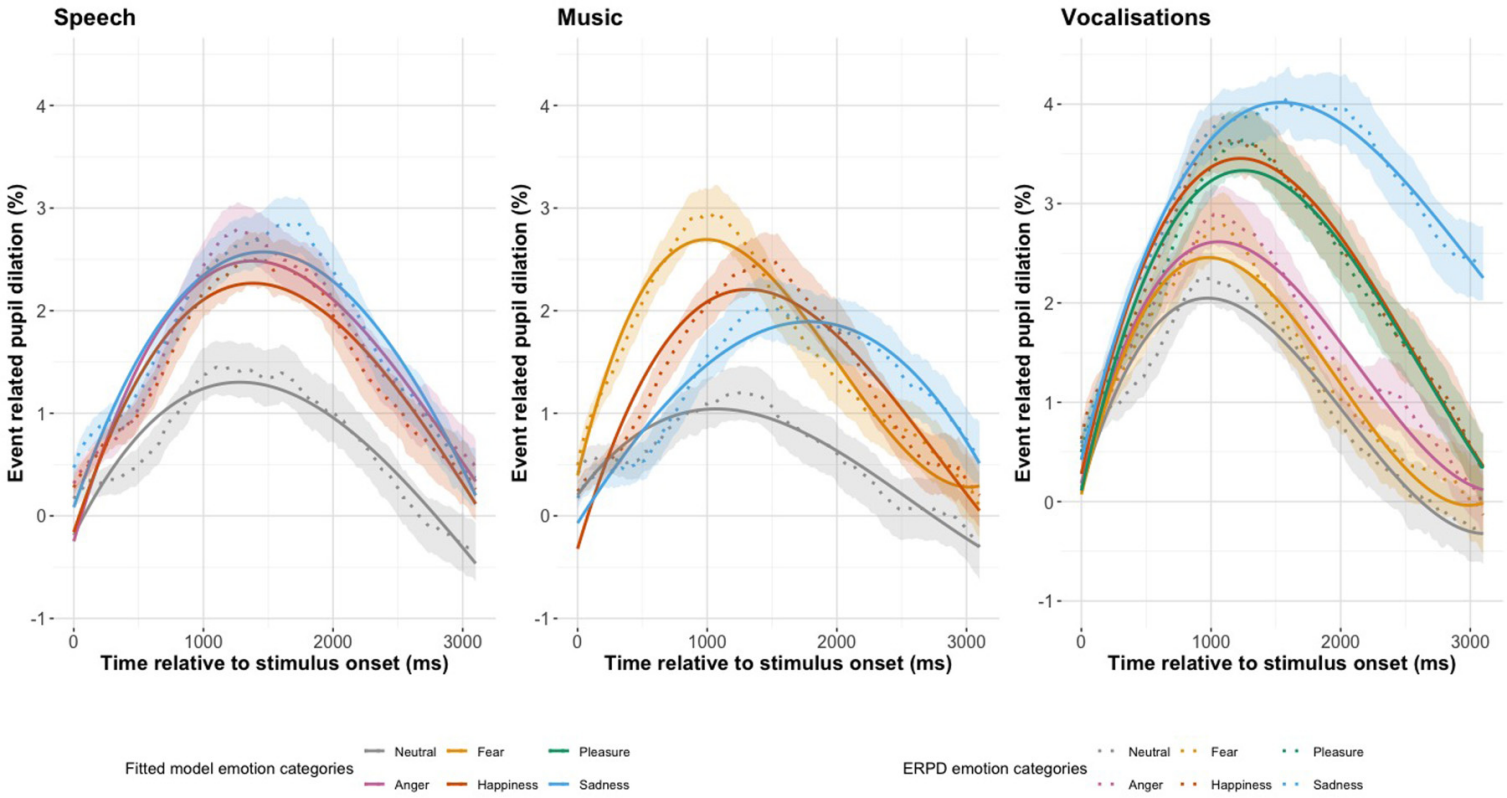
The Change in Pupil Dilation, as Reflected by the Event Related Pupil Dilation (ERPD) and the Fitted Model Values for Emotion Category in Each Stimulus Type of Pseudospeech, Music, and Vocalizations. The Shaded Interval Indicates the 95% Confidence Interval of the Pupil Dilation Curves.

Figure 3 shows the ERPDs with 95% confidence intervals and the fitted GCA model values for pseudospeech, music, and vocalizations (Supplemental material, Tables 4, 5 and 7). In Figure 3(Speech) we see the pseudospeech stimulus type for the four emotion categories; neutral, anger, happiness, and sadness. The overall shape of the ERPDs in Figure 3(Speech) is similar for all four categories, showing an increase from baseline, peaking around 1500 ms and then returning to baseline. We see that the three categories of anger, happiness, and sadness reach a peak that is almost double in size of the neutral category. In Figure 3(Music) we see the music stimulus type for the four emotion categories: neutral, fear, happiness, and sadness. The overall shape of the ERPDs in Figure 3(Music) are generally different from each other. The fear category has a large and early peak, and sadness has a smaller and later peak, with happiness somewhere in between. The neutral category has the smallest peak pupil dilation. In Figure 3(Vocalizations) we see the vocalization stimuli type for the six emotion categories: neutral, fear, anger, happiness, pleasure, and sadness. The neutral category peaks higher than the pseudospeech and music ERPDs. The sadness category has a large and late peak. Happiness and pleasure have the second largest peak dilation and both follow a very similar pattern.

Research Question 1: Is emotion processing reflected in the pupillary response to emotional pseudosentences?

#### Pseudospeech

Table 1 shows the summary model estimates and significance codes for the pseudospeech emotion category model, the full model estimates and standard errors can be found in the Supplemental material, Table 4(a). The full model included a fixed effect of emotion, χ^2^(3) = 13.8, *p* = .003, and emotion as an interaction on the quadratic, χ^2^(3) = 9.7, *p* = .021, term of the polynomial. Neutral was included in the intercept, and random effects of subject on the linear, quadratic, and cubic terms, as well as subject-by-emotion on the linear, quadratic, and cubic terms were included. In Figure 3(Speech), we see that the emotion categories have a greater average dilation than the neutral category, and this was reflected in the intercept of our GCA model (Anger: Est = 0.783, SE = 0.243, *p* = .001; Happiness: Est = 0.662, SE = 0.243, *p* = .006; Sadness: Est = 0.849, SE = 0.243, *p* < .001). The sharpest peak dilation, as reflected by the quadratic time term of the polynomial, was observed for the anger category (Est = −3.797, SE = 1.277, *p* = .003). The addition of the linear time term as an interaction on emotion did not improve model fit, χ^2^(3) = 2.6, *p* = .463, nor did the addition of the cubic term, χ^2^(3) = 5.6, *p* = .133, indicating that differences between emotional categories were captured in the magnitude and symmetry of dilation, rather than the time course of shape of the curve.

**Table 1.** Summary Model Estimates for the Pseudospeech Emotion Category Model, Showing the Interaction of Each Emotion Category With Each Time Term.

	Intercept	Quadratic
Intercept	0.762	−6.112 (*)
Anger	0.783 (*)	−3.797 (*)
Happiness	0.663 (*)	−3.111 (*)
Sadness	0.849 (*)	−3.309 (*)

Model formula: erpd ∼ Linear + Cubic + Quadratic * Emotion + (Linear + Quadratic + Cubic | Subject:Emotion) + (Linear + Quadratic + Cubic | Subject). *p < .05. Total number of observations: 147,258, number of subjects: 21, number of subject:emotion: 84.

Research Question 2. How does the pupillary response to emotional pseudosentences compare to non-word human vocalizations and instrumental music?

#### Music

Table 2 shows the summary model estimates and significance codes for the music emotion category model, the full model estimates and standard errors can be found in the Supplemental material, Table 5(a). Our final model included fixed effects of emotion, χ^2^(3) = 13.1, *p* = .004, and an interaction of emotion on the linear, χ^2^(3) = 20.2, *p* < .001; quadratic χ^2^(3) = 19.3, *p* < .001; and cubic time term of the polynomial, χ^2^(3) = 34.0, *p* *<* .001. Neutral was included in the intercept, and random effects of subject on the linear, quadratic, and cubic time term were included, as well as subject-by-emotion on the linear, quadratic, and cubic time term. There was an effect of emotion category on the intercept of our model, indicating that each emotion had a significant greater average ERPD than neutral (Fear: Est = 1.026, SE = 0.294, *p* < .001; Happiness: Est = 0.882, SE = 0.294, *p* = .003; Sadness: Est = 0.745, SE = 0.294*, p* = .011). The largest overall dilation was observed for fear, which peaked early and had a steep decline, as reflected in the negative linear time term (Est = –3.595, SE = 1.154, *p* = .054). In Figure 3 (Music) we can see how sadness peaks much later than neutral, fear and happiness, this is reflected in the linear time term, which showed that the sadness category (Est = 5.394, SE = 1.866, *p* = .004) had the greatest overall slope of the pupillary curve. The curve with the most symmetricity around the peak, as reflected by the quadratic time term, was observed for the happiness category (Est = −5.449, SE = 0.160, *p* < .001). The curve with the sharpest rise and fall, reflected by the cubic time term, is seen for the fear category (Est = 3.818, SE = 0.806, *p* < .001).

**Table 2.** Summary Model Estimates and Significance Codes for the Music Emotion Category Model, Showing the Interaction of Each Emotion Category With Each Time Term.

	Intercept	Linear	Quadratic	Cubic
Intercept	0.473	−3.744	−3.592 (*)	0.904
Fear	1.026 (*)	−3.595	−3.242 (*)	3.818 (*)
Happiness	0.882 (*)	1.429	−5.449 (*)	1.195
Sadness	0.745 (*)	5.394 (*)	−3.191 (*)	−1.390

Model formula: erpd ∼ (Linear + Quadratic + Cubic) * Emotion + (Linear + Quadratic + Cubic| Subject) + (Linear + Quadratic + Cubic | Subject:Emotion). *p < .05. Total number of observations: 173,988, number of subjects: 21, number of subject:emotion: 84.

A *post hoc* analysis revealed differences between some emotion categories on different time terms of the polynomial. The full results of the *post hoc* tests can be found in the Supplemental material, Table 6. There were differences in the cubic time term for fear with each other emotion (fear vs. neutral: z-value = –4.735, *p* < .001; fear vs. happiness: z value = –3.257, *p* = .023; fear vs. sadness: z value = –6.451, *p* < .001) and differences with happiness vs. sadness: z value = –3.207, *p* = .027. The quadratic time term revealed only one significant pairwise comparison, neutral vs. happiness: z value = −4.722, *p* < .001. The linear time term confirmed the delayed ERPD peak in sadness vs. fear: z value = 4.817, *p* < .001. The average ERPD response revealed differences in fear vs. neutral: z value = –3.495, *p* = .0104 and near differences in neutral vs. happiness: z value = 3.006, *p* = .050.

#### Vocalizations

Table 3 shows the summary model estimates and significance codes for the vocalizations emotion category model, the full model estimates and standard errors can be found in the Supplemental material, Table 7(a). Our full model included fixed effects of emotion, χ^2^(5) = 35.8, *p* < .001, and an interaction of emotion on the linear time term, χ^2^(5) = 21.4, *p* = .001 and the quadratic time term, χ^2^(5) = 29.4, *p* < .001. Neutral was included in the intercept, and random effects of subject on all time terms of the polynomial, as well as subject-by-emotion on all time terms of the polynomial were included. The pupillary curve with the greatest intercept, and therefore the greatest area under the curve, was observed for the sadness stimuli (Est = 2.05, SE = 0.367, *p* < .001). In Figure 3(Vocalizations), we see how each of the emotion categories peaks and declines similarly, except for the sadness category, which has a wider peak and slow decline. This observation is reflected in the interaction with emotion on the linear time term, which shows the high model estimate value for the sadness stimuli (Est = 8.25, SE = 1.927, *p* < .001). The emotion curve with the greatest symmetry around the peak, as reflected by the quadratic time term, was observed for the happiness category (Est = –5.8231, SE = 1.402, *p* < .001).

**Table 3.** Summary Model Estimates and Significance Codes for the Full Vocalizations Emotion Category Model, Showing the Interaction of Each Emotion Category With Each Time Term.

	Intercept	Linear	Quadratic
Intercept	1.109 (*)	3.608 (*)	−6.787 (*)
Anger	0.400	0.885	−1.590
Fear	0.056	0.598	−0.172
Happiness	1.206 (*)	1.199	−5.823 (*)
Pleasure	1.018 (*)	1.892	−5.369 (*)
Sadness	2.051 (*)	8.24 (*)	−4.715 (*)

Full model: erpd ∼ (Linear + Quadratic) * Emotion + Cubic + (Linear + Quadratic + Cubic | Subject:Emotion) + (Linear + Quadratic + Cubic | Subject). *p < .05. Total number of observations: 168,804, number of subjects: 21, number of subject:emotion: 158.

Fear and anger, by contrast, did not differ significantly from neutral on any time term. The interaction of emotion on the cubic time term of the polynomial did not improve model fit χ^2^(5) = 10.396, *p* = .065. This indicates that the rise and fall of the pupillary curves did not differ between emotions, this is reflected in Figure 3(Vocalizations), as we see that each of the emotion curves have similar timing in their peak dilations.

A *post hoc* analysis revealed differences between some emotion categories on different time terms of the polynomial. The full results of the *post hoc* tests can be found in the Supplemental material, Table 8. For the quadratic time term, fear had differences with three emotion categories (fear vs. pleasure: z value = 3.709, *p* < .01; fear vs. happiness: z value = –4.209, *p* < .01; fear vs. sadness: z value = –3.242, *p* = .043) and neutral revealed the same differences as with fear (neutral vs. pleasure: z value = 3.833, *p* < .01; neutral vs. happiness: z value = –4.153, *p* < .01; neutral vs. sadness: z value = –3.366, *p* = .029). In the linear time term, sadness revealed significant differences with every other emotion category (sadness vs. neutral: z value = 4.281, *p* < .001; sadness vs. fear: z value = 3.969, *p* < .01; sadness vs. anger: z value = 3.821, *p* < .01; sadness vs. pleasure: z value = 3.299, *p* < .036; sadness vs. happiness: z value = 3.654, *p* = .010). The average ERPD response revealed differences for three sadness comparisons (sadness vs. neutral: z value = 5.591, *p* < .001; sadness vs. fear: z value = 5.438, *p* = <.001; sadness vs. anger: z value = 4.501, *p* < .001) and neutral vs. happiness: z value = 3.287, *p* = .038.

**Figure 4.**
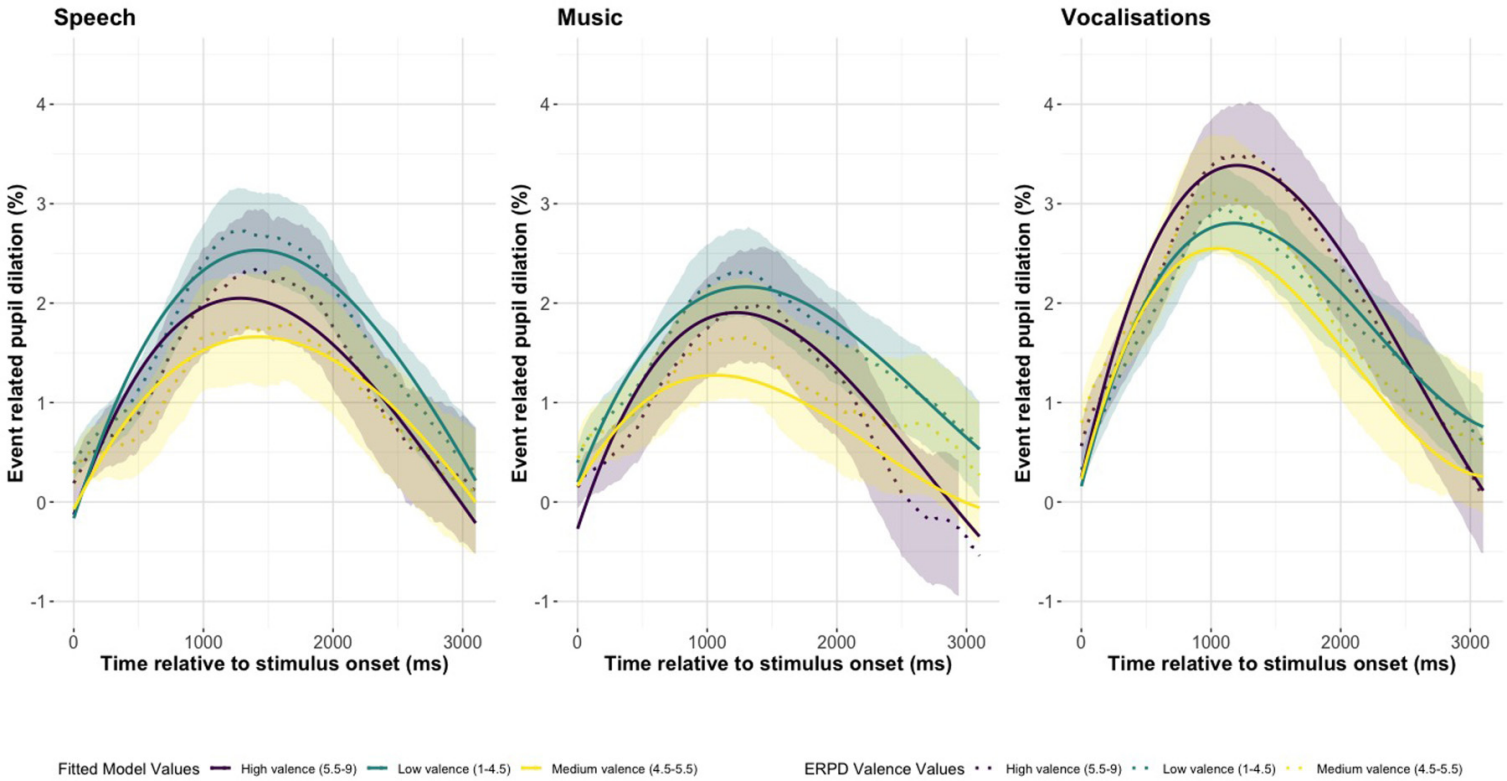
The Change in Pupil Dilation, as Reflected by the Event Related Pupil Dilation (ERPD) and the Fitted Model Values for Valence in Each Stimulus Type of Pseudospeech, Music, and Vocalizations. The Shaded Interval Indicates the 95% Confidence Interval of the Pupil Dilation Curves.

**Figure 5.**
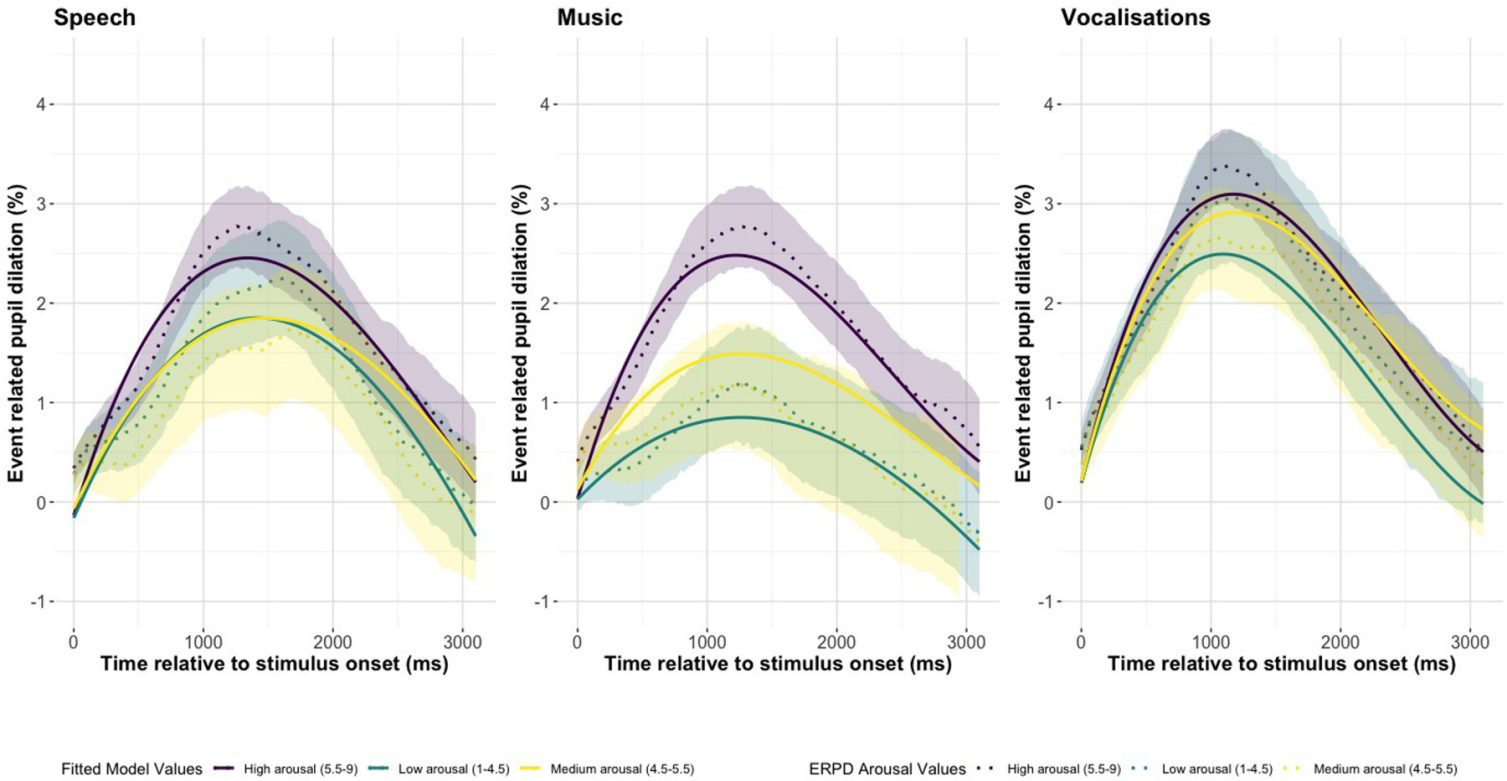
The Change in Pupil Dilation, as Reflected by the Event Related Pupil Dilation (ERPD) and the Fitted Model Values for Arousal in Each Stimulus Type of Pseudospeech, Music, and Vocalizations. The Shaded Interval Indicates the 95% Confidence Interval of the Pupil Dilation Curves.

Figures 4 and 5 show the valence and arousal fitted model values and the ERPDs with 95% confidence intervals pseudospeech, music, and vocalizations. In Figure 5 we see that high arousal results in overall larger pupillary curves in pseudospeech, music, and vocalizations. Figure 5(Music) shows the strongest distinction between high, medium, and low arousal. In Figure 4, the high and low valence ERPDs are all greater than those with medium valence, with low valence having the greatest ERPD in Figure 4(Speech) and Figure 4(Music), but high valence has the largest pupil response in vocalizations.

#### Pseudospeech, Music, and Vocalizations

Table 4 shows the full model estimates and standard errors for the valence and arousal stimuli comparison model. The random effect variance estimates can be found in the Supplemental Table 9. We tested the effect of stimuli type, valence, and arousal on ERPD. Our full model included a three-way interaction of the quadratic term and stimulus type on valence, χ^2^(2) = 73.002, *p* < .001, and arousal, χ^2^(2) = 38.666, *p* < .001. The model included interactions of valence with the linear, χ^2^(2) = 43.881, *p* < .001, and cubic term of the polynomial, χ^2^(2) = 71.602, *p* < .001. Pseudospeech was included in the intercept, and random effects of subject on the linear, quadratic, and cubic time terms, as well as item on the linear, quadratic, and cubic time terms were included.

**Table 4.** Full Model Estimates and Standard Errors for the Valence and Arousal Stimuli Model.

	Estimate	SE	*t* value	*p*	Sig
(Intercept)	1.402	0.417	3.364	<.001	*
Linear time term	−3.228	2.057	−1.570	0.167	
Quadratic time term	−8.763	1.358	−6.454	<.001	*
Cubic time term	2.249	0.643	2.496	<.001	*
Valence	−0.137	0.010	−14.075	<.001	*
Arousal	0.091	0.010	9.292	<.001	*
Stimulus Music	−0.228	0.170	−1.347	0.178	
Stimulus Vocalizations	0.397	0.171	2.322	0.020	*
Linear term:Valence	−1.459	0.064	−22.694	<.001	*
Quadratic term:Valence	0.645	0.117	5.527	<.001	*
Quadratic term:Arousal	−0.199	0.115	−1.735	0.083	
Cubic term:Valence	0.318	0.056	5.627	<.001	*
Quadratic term:Music	2.241	0.827	2.709	0.007	*
Quadratic term:Vocalizations	−0.281	0.834	−0.337	0.736	
Valence:Music	−0.090	0.013	−7.111	<.001	*
Valence:Vocalizations	0.170	0.014	12.292	<.001	*
Arousal:Music	−0.019	0.013	−1.479	0.139	
Arousal:Vocalizations	0.045	0.013	3.523	<.001	*
Quadratic term:Valence:Music	−0.245	0.152	−1.614	0.107	
Quadratic term:Valence:Vocalizations	−1.358	0.160	−8.464	<.001	*
Quadratic term:Arousal:Music	−0.543	0.149	−3.640	<.001	*
Quadratic term:Arousal:Vocalizations	−0.672	0.154	−4.371	<.001	*

Full model formula: erpd ∼ Quadratic * Stimulus Type * (Valence + Arousal) + (Linear + Cubic) * Valence + (Linear + Quadratic + Cubic | Subject) + (Linear + Quadratic + Cubic | Item). * *p* < .05. Total number of observations: 490,050, number of subject: 21, number of items: 158.

There was a significant interaction of valence with the linear, Est = –1.459, SE = 0.064, *p* < .001, quadratic, Est = 0.645, SE = 0.117, *p* < .001, and cubic time term, Est = 0.318, SE = 0.056, *p* <.001. The large negative effect of valence on the linear term reflects a sharp decrease in the pupil at earlier time points. Valence had significant interactions with stimulus (Music: Est = –0.090, SE = 0.013, *p* < .001; Vocalizations: Est = 0.16, SE = 0.014, *p* < .001) and three-way interactions with vocalizations on the quadratic term, Est = –1.358, SE = 0.160, *p* < .001. Valence had a strong effect on the quadratic term in vocalizations, which reflects the curvature of the pupil around the peak. There was a significant interaction of arousal with vocalizations, Est = 0.045, SE = 0.013, *p* < .001, and in a three-way interaction with the quadratic time term, Est = –0.672, SE = 0.154, *p* < .001. There was a significant three-way interaction of arousal with music on the quadratic term, Est = –0.543, SE = 0.149, *p* < .001, and a significant positive interaction of vocalizations with valence, Est = 0.170, SE = 0.014, *p* < .001, reflecting that the average ERPD increases for vocalizations by valence.

## Discussion

The current study examined the use of pupil dilation as a measure of auditory emotion processing. We identified that emotional pseudosentences elicit an overall higher average pupil dilation than neutral, in combination with a faster rise and fall in the time course of the pupil, thus confirming that pupil dilation can index emotion processing in pseudospeech. A similar effect was seen in music, with more nuances in the time course of pupil dilation. Although fear and anger in vocalizations failed to evoke a significant difference from neutral, the other emotions all showed increases in dilation compared with neutral. Vocalizations resulted in overall higher average dilations than pseudospeech, and average dilations in music did not differ from pseudospeech. We also report that valence has an overall stronger impact on pupil dilation than arousal. Our results are in line with previous research, showing that the pupillary response results in increased dilations for emotional speech, music, and human vocalizations (Gingras et al., 2015; Jürgens et al., 2018; Oliva & Anikin, 2018). The pupillary response to pseudospeech was not sensitive enough to distinguish between emotion categories, however, the time course of the pupillary response to music reflected differences between emotion categories.

### Pseudospeech

Our results are the first to indicate that purely prosodic emotional pseudospeech triggers increased pupil dilations. This index in pupil dilation is likely a reflection of emotion processing, rather than cognitive, as participants showed no differences in recognition rates between emotion categories and neutral. Low valence has a strong impact on the pupillary response, with sadness producing the greatest average pupil dilation, and anger the strongest effect on the symmetricity of the peak pupil dilation. We did not see differences in the time course of the pupil between emotions in our pseudospeech stimuli, which is in line with research showing that the pupil might not be sensitive enough to delineate between emotions expressed in speech (Partala & Surakka, 2003), which has also been observed in semantically neutral speech (Kuchinke et al., 2011). Although Jürgens et al. (2018) showed that pupil responses to angry speech stimuli dilated faster, and the pupil response to joyful speech was delayed by 1 s. In addition, in poetic language, the pupil can differentiate between semantic comical and aesthetical poetry that were presented in a neutral tone (Niikuni et al., 2022). This shows that the pupil might be sensitive enough to distinguish between emotion categories for semantic speech stimuli, further research in pupil dilation studies should look at combining both semantic and prosodic emotion information.

### Music

Pupil responses to music varied over time depending on the emotion. Fear evoked an early and strong response, likely a reflection of autonomic arousal (Mathôt, 2018), and in line with research that highly arousing music induces increased pupil dilation compared to low arousal music (Gingras et al., 2015). Sadness evoked a slower, gradual increase, likely indicative of cognitive and emotional processing. These differences can be explained by stimulus characteristics: the MEB fear stimuli were portrayed by a high-pitched note, whereas sadness was expressed as a short minor melody. However, our stimuli were not controlled for duration, and the fear stimuli were on average 1 s while the sadness stimuli were more than 2 s. Pupil dilation is sensitive to the duration of the stimulus and this can explain the differences we observed between emotions (Oliva & Anikin, 2018). Duration is inherent to certain emotions, sadness tends to unfold more slowly than fear or anger (Yildirim et al., 2004), it is an acoustic pattern that is a part of the emotion and future studies should account for these acoustic-emotional links.

### Vocalizations

The greatest average pupillary response was observed for sadness, which showed an extended peak dilation without a return to baseline in the analysis time window. This sustained dilation to sadness could be related to the social relevance of cries, thus eliciting a stronger and more sustained emotional response than to that of laughter (Cosme et al., 2021). In contrast, high-valence emotions, like happiness and pleasure, evoked sharp and symmetric peak pupil dilations. Previous research has shown mixed results as to whether positively and negatively valence emotions differentially affect pupil size (Bradley et al., 2008; Jürgens et al., 2018; Kuchinke et al., 2011). Our results found that anger and fear did not differ significantly from neutral on any of the time components of the pupillary curve. This could be related to duration; anger, fear, and neutral had the shortest durations of all the categories. It could also reflect the confidence of participants’ ratings to human vocalizations, and anger and fear were less easily recognized compared to neutral, happiness, and sadness. But this lack of distinction from neutral could also be explained by emotion processing, as both anger and fear were rated as having less negative valence than sadness, and pupils respond to the intensity of the valence of vocalizations, with less intense stimuli producing smaller dilations (Oliva & Anikin, 2018). Their results also showed percentage change in pupil response ranging from 3% to 13%, where a low percentage change occurred when the relative baseline was high. Our ERPD results showed percentage pupillary changes around 3%–4%, which could be related to a high relative baseline pupil dilation in our participants.

### Limitations

The pupil is a known indicator of autonomic activation, attention, and cognitive processing (Granholm & Steinhauer, 2004; Hess & Polt, 1964). In our experiment, participants listened to emotional auditory stimuli as we recorded their pupil dilations, and then performed an emotion recognition task. The pupillary responses observed likely reflect a combination of both autonomic activation and cognitive processing, as participants were required to rate the stimuli. This combination is inherent in our stimuli sets, where vocalizations are an indicator of autonomic arousal, and pseudospeech and music require more processing of information to recognize an emotion. Our study included six emotion categories for human vocalizations, four for music, and four for pseudospeech. While emotions in vocalizations are generally easier to recognize than speech (Hawk et al., 2009) and vocalizations had similar or higher rates of recognition than music and pseudospeech in our results, the larger stimulus set in vocalizations could have contributed to differences in pupillary responses arising from task difficulty. Future studies would benefit from including a number of additional measures such as subjective difficulty ratings and both a passive and active-listening task to further disentangle the mechanisms underlying pupil responses. While we intentionally broadened the context by comparing pseudospeech, music, and vocalizations, we acknowledge that fully disentangling these mechanisms may not be entirely possible.

GCA was used to analyze the ERPD results (Mirman, 2014). While this is a powerful method and can simplify interpretation by analyzing the pupil on different time curves, the method does not account for changes in gaze position or autocorrelation of pupil dilation. Other methods such as generalized additive mixed modelling (van Rij et al., 2019) and the temporal response function (Crosse et al., 2016) provide built-in methods to handle autocorrelation of the dependent variable. Future studies would benefit from using methods to handle autocorrelation of the pupil signal for more robust results. Our participants rated valence and arousal on a scale of 1–9, and could choose any number in between the whole numbers, up to three decimal places. We therefore needed to treat this data as continuous, but this would have been more suited as an ordinal scale. These ratings represent a subjective distance that varies between participants, in future studies this data should include a fixed whole number Likert scale that is then treated as ordinal.

### Applications

Pupil dilation is known to reflect both cognitive and emotional processing, and does not reflect explicit emotion recognition, but its sensitivity to emotional salience offers insight into underlying emotion perception. This insight may benefit different populations with known difficulties in emotion recognition such as autism, hearing loss, and second language speakers (Christensen et al., 2019; Goy et al., 2018; Iacozza et al., 2017; Kuchinke et al., 2011; Parbery-Clark et al., 2013). This research could aid in improving hearing aids and cochlear implants for emotion recognition, by using pupillometry as a tool to measure emotion perception in speech, as well as short musical and human vocalization excerpts. Individuals with autism focus on semantics when trying to understand emotions in speech (Dennis et al., 2000), whereas individuals with hearing loss have increased effort to understand speech. The applicability of pseudosentences in testing these populations provides a clear stimulus to test emotion processing of speech prosody. The intensity of the valence/arousal of the stimulus, as we’ve shown, is not the only factor in modulating pupil dilation, the type of sound stimulus is also a factor. Affective stimuli corpora often include a range of affective stimuli types, from crying babies, to animal noises, to environmental sounds (Parsons et al., 2014; Yang et al., 2018), and future studies should take into account the effect of stimulus type when measuring auditory emotion processing.

## Supplemental Material

sj-docx-1-tia-10.1177_23312165251365824 - Supplemental material for The Time Course of the Pupillary Response to Auditory Emotions in Pseudospeech, Music, and Vocalizations Supplemental material, sj-docx-1-tia-10.1177_23312165251365824 for The Time Course of the Pupillary Response to Auditory Emotions in Pseudospeech, Music, and Vocalizations by Julie Kirwan, Deniz Başkent and Anita Wagner in Trends in Hearing
