# Supplementary material for: The Time Course of the Pupillary Response to Auditory Emotions in Pseudospeech, Music, and Vocalizations
Source: Trends Hear. 2025 Aug 10;29:23312165251365824. doi: 10.1177/23312165251365824 (PMC12340197; doi:10.1177/23312165251365824)
Supplement: sj-docx-1-tia-10.1177_23312165251365824 - Supplemental material for The Time Course of the Pupillary Response to Auditory Emotions in Pseudospeech, Music, and Vocalizations [file sj-docx-1-tia-10.1177_23312165251365824.docx]

**Supplementary material**

*Supplementary Table 1(a). GLMER model estimates for the full model to test the fixed and interaction effects of emotion category and stimulus type on emotion recognition scores. Model formula = glmer(score ~ Emotion * Stimulus + (1 + Emotion | Subject) + (1 | Item), family='binomial'). OR=Odds Ratio *=<0.05. Speech and neutral in the intercept. Total number of observations: 2948, number of subjects: 21, number of items: 156*

|  | Estimate | SE | OR | *Z* value | *p* | Sig | |
| --- | --- | --- | --- | --- | --- | --- | --- |
| (Intercept) | 2.440 | 0.473 | 11.473 | 5.156 | <0.001 | * |  |
| Emotion Anger | -0.112 | 0.661 | 0.894 | -0.169 | 0.8657 |  |  |
| Emotion Fear | -1.773 | 0.773 | 0.170 | -2.294 | 0.0218 | * |  |
| Emotion Happiness | -0.949 | 0.600 | 0.387 | -1.582 | 0.1136 |  |  |
| Emotion Pleasure | -2.340 | 0.766 | 0.096 | -3.054 | 0.0023 | * |  |
| Emotion Sadness | -0.539 | 0.627 | 0.584 | -0.859 | 0.3901 |  |  |
| Stimulus Music | -1.093 | 0.528 | 0.335 | -2.069 | 0.0386 | * |  |
| Stimulus Vocalisations | 1.285 | 0.671 | 3.615 | 1.915 | 0.0555 | . |  |
| Fear:Music | 1.945 | 0.873 | 6.992 | 2.228 | 0.0259 | * |  |
| Happy:Music | 2.416 | 0.764 | 11.199 | 3.161 | 0.0016 | * |  |
| Sadness:Music | 1.000 | 0.753 | 2.717 | 1.327 | 0.1844 |  |  |
| Anger:Vocalisations | -1.696 | 0.923 | 0.183 | -1.838 | 0.0660 | . |  |
| Happiness:Vocalisations | 3.021 | 1.345 | 20.507 | 2.246 | 0.0247 | * |  |
| Sadness:Vocalisations | 0.856 | 0.993 | 2.353 | 0.861 | 0.3891 |  |  |

*Supplementary Table 1(b). GLMER model estimates for the full model to test the fixed and interaction effects of emotion category and stimulus type on emotion recognition scores. Model formula = glmer(score ~ Emotion * Stimulus + (1+Emotion|subject) + (1|item), family='binomial'). *=<0.05. Vocalisations and sadness in the intercept. Total number of observations: 2948, number of subjects: 21, number of items: 156*

|  | Estimate | SE | *Z* value | *p* | Sig |
| --- | --- | --- | --- | --- | --- |
| (Intercept) | 4.0421 | 0.633 | 6.383 | <0.001 | * |
| Emotion Neutral | -0.317 | 0.868 | -0.365 | 0.715 |  |
| Emotion Anger | -2.125 | 0.799 | -2.661 | 0.00779 | * |
| Emotion Fear | -2.090 | 0.775 | -2.696 | 0.00701 | * |
| Emotion Happy | 1.755 | 1.262 | 1.391 | 0.16427 |  |
| Emotion Pleasure | -2.658 | 0.827 | -3.214 | 0.00131 | * |
| Stimulus Speech | -2.141 | 0.732 | -2.923 | 0.00347 | * |
| Stimulus Music | -2.234 | 0.712 | -3.137 | 0.00171 | * |
| Neutral:Speech | 0.856 | 0.992 | 0.862 | 0.38850 |  |
| Anger:Speech | 2.552 | 0.968 | 2.637 | 0.00837 | * |
| Happy:speech | -2.165 | 1.375 | -1.575 | 0.11531 |  |
| Neutral:Music | -0.144 | 0.961 | -0.15 | 0.88075 |  |
| Fear:Music | 1.801 | 0.921 | 1.955 | 0.05061 | . |
| Happy:Music | -0.749 | 1.365 | -0.549 | 0.58313 |  |

*Supplementary Table 1(c). GLMER model estimates for the full model to test the fixed and interaction effects of emotion category and stimulus type on emotion recognition scores. Model formula = glmer(score ~ Emotion * Stimulus + (1+Emotion|subject) + (1|item), family='binomial'). *=<0.05. Vocalisations and happiness in the intercept. Total number of observations: 2948, number of subjecst: 21, number of items: 156*

|  | Estimate | SE | *Z* value | *p* | Sig |
| --- | --- | --- | --- | --- | --- |
| (Intercept) | 5.797 | 1.109 | 5.225 | <0.001 | * |
| Emotion Neutral | -2.072 | 1.250 | -1.657 | 0.09751 | . |
| Emotion Anger | -3.879 | 1.211 | -3.204 | 0.00136 | * |
| Emotion Fear | -3.845 | 1.1968 | -3.212 | 0.00132 | * |
| Emotion Pleasure | -4.412 | 1.225 | -3.601 | <0.001 | * |
| Emotion Sad | -1.755 | 1.261 | -1.391 | 0.16420 |  |
| Stimulus Speech | -4.306 | 1.165 | -3.697 | <0.001 | * |
| Stimulus Music | -2.983 | 1.166 | -2.558 | 0.01054 | * |
| Neutral:Speech | 3.021 | 1.342 | 2.250 | 0.02442 | * |
| Anger:Speech | 4.717 | 1.326 | 3.557 | <0.001 | * |
| Sad:speech | 2.165 | 1.375 | 1.575 | 0.11528 |  |
| Neutral:Music | 0.605 | 1.333 | 0.454 | 0.64992 |  |
| Fear:Music | 2.550 | 1.305 | 1.954 | 0.05070 | . |
| Sad:Music | 0.749 | 1.365 | 0.549 | 0.58313 |  |

*Supplementary Table 1(d). GLMER model estimates for the full model to test the fixed and interaction effects of emotion category and stimulus type on emotion recognition scores. Model formula = glmer(score ~ Emotion * Stimulus + (1+Emotion|subject) + (1|item), family='binomial'). *=<0.05. Music and fear in the intercept. Total number of observations: 2948, number of subjecst: 21, number of items: 156*

|  | Estimate | SE | *Z* value | *p* | Sig |
| --- | --- | --- | --- | --- | --- |
| (Intercept) | 1.519 | 0.393 | 3.867 | <0.001 | * |
| Emotion Neutral | -0.172 | 0.588 | -0.293 | 0.76986 |  |
| Emotion Anger | -0.035 | 0.680 | -0.051 | 0.95903 |  |
| Emotion Happy | 1.294 | 0.557 | 2.323 | 0.02018 | * |
| Emotion Pleasure | -0.568 | 0.699 | -0.811 | 0.41709 |  |
| Emotion Sad | 0.289 | 0.527 | 0.548 | 0.58344 |  |
| Stimulus Speech | 0.093 | 0.537 | 0.174 | 0.86206 |  |
| Stimulus Vocalisations | 0.433 | 0.586 | 0.740 | 0.45952 |  |
| Neutral:Speech | 0.999 | 0.753 | 1.327 | 0.18447 |  |
| Anger:Speech | 0.751 | 1.015 | 0.740 | 0.45919 |  |
| Happy:speech | -1.416 | 0.771 | -1.836 | 0.06629 | . |
| Neutral:Vocalisations | 1.945 | 0.873 | 2.228 | 0.02591 | * |
| Happy:Vocalisations | 2.550 | 1.307 | 1.951 | 0.05108 | . |
| Sad:Vocalisations | 1.801 | 0.922 | 1.952 | 0.05091 | . |

*Supplementary Table 1(e). GLMER model estimates for the full model to test the fixed and interaction effects of emotion category and stimulus type on emotion recognition scores. Model formula = glmer(score ~ Emotion * Stimulus + (1+Emotion|subject) + (1|item), family='binomial'). *=<0.05. Speech and anger in the intercept. Total number of observations: 2948, number of subjecst: 21, number of items: 156*

|  | Estimate | SE | *Z* value | *p* | Sig |
| --- | --- | --- | --- | --- | --- |
| (Intercept) | 2.328 | 0.446 | 5.217 | <0.001 | * |
| Emotion Neutral | 0.112 | 0.660 | 0.169 | 0.86568 |  |
| Emotion Fear | 0.035 | 0.679 | 0.051 | 0.95904 |  |
| Emotion Happy | -0.837 | 0.591 | -1.417 | 0.15650 |  |
| Emotion Pleasure | -0.533 | 0.733 | -0.726 | 0.46770 |  |
| Emotion Sad | -0.427 | 0.598 | -0.714 | 0.47510 |  |
| Stimulus Music | -0.093 | 0.537 | -0.174 | 0.86198 |  |
| Stimulus Vocalisations | -0.411 | 0.632 | -0.651 | 0.51519 |  |
| Neutral:Music | -0.999 | 0.753 | -1.328 | 0.18427 |  |
| Fear:Music | -0.751 | 1.014 | -0.741 | 0.45877 |  |
| Happy:Music | 1.416 | 0.771 | 1.837 | 0.06615 | . |
| Neutral:Vocalisations | 1.696 | 0.922 | 1.839 | 0.06586 | . |
| Happy:Vocalisations | 4.717 | 1.329 | 3.550 | <0.001 | * |
| Sad:Vocalisations | 2.552 | 0.968 | 2.635 | 0.00841 | * |

*Supplementary Table 1(f). Random effect variance estimates for the emotion recognition score model described in Supplementary Table 1(a-e)*

| Group | Effect | Variance | *SD* | *Correlation* |  |  |  | |  |
| --- | --- | --- | --- | --- | --- | --- | --- | --- | --- |
| Item | Intercept | 1.285 | 1.133 |  |  |  | |  |  |
| Subject | Intercept | 1.163 | 1.078 |  |  |  | |  |  |
| Subject | Anger | 1.869 | 1.367 | -0.90 |  |  | |  |  |
| Subject | Fear | 1.635 | 1.279 | -0.90 | 0.89 |  | |  |  |
| Subject | Happiness | 0.888 | 0.942 | -0.88 | 0.89 | 0.93 | |  |  |
| Subject | Pleasure | 1.369 | 1.170 | -0.55 | 0.41 | 0.51 | | 0.30 |  |
| Subject | Sadness | 1.295 | 1.138 | -0.90 | 0.87 | 0.93 | | 1.00 | 0.31 |

*Supplementary Table 2(a). Model estimates of the full valence model to test the fixed and interaction effects of stimulus type and emotion category on valence ratings: Formula = Valence ~ Stimulus * Emotion + (1 + Emotion + Stimulus | Subject) + (1 | Item). *=<0.05. Total number of observations: 2948, number of subjects: 21, number of items: 156*

|  | Estimate | SE | *t* value | *p* | Sig |
| --- | --- | --- | --- | --- | --- |
| (Intercept) | 5.135 | 0.126 | 40.639 | <0.001 | * |
| Stimulus Vocalisations | -0.161 | 0.188 | -0.858 | 0.4097 |  |
| Stimulus Music | -0.397 | 0.181 | -2.196 | 0.0295 | * |
| Emotion Anger | -1.798 | 0.229 | -7.867 | <0.001 | * |
| Emotion Fear | -1.525 | 0.242 | -6.313 | <0.001 | * |
| Emotion Happiness | 1.381 | 0.216 | 6.930 | <0.001 | * |
| Emotion Pleasure | 1.178 | 0.240 | 4.913 | <0.001 | * |
| Emotion Sadness | -1.161 | 0.214 | -7.539 | <0.001 | * |
| Vocalisations:Anger | -0.107 | 0.267 | -0.401 | 0.6893 |  |
| Music:Fear | 0.148 | 0.257 | 0.577 | 0.5650 |  |
| Vocalisations:Happiness | 1.151 | 0.263 | 4.370 | <0.001 | * |
| Music:Happiness | 0.949 | 0.235 | 4.033 | <0.001 | * |
| Vocalisations:Sadness | -1.004 | 0.263 | -3.882 | <0.001 | * |
| Music:Sadness | 0.294 | 0.235 | 1.254 | 0.2117 |  |

*Supplementary Table 2(b) Random effect variance estimates for the valence model described in Supplementary Table 2(a).*

| Group | Effect | Variance | *SD* | *Correlation* |  |  |  | |  |  |  |
| --- | --- | --- | --- | --- | --- | --- | --- | --- | --- | --- | --- |
| Item | Intercept | 0.232 | 0.481 |  |  |  | |  |  |  |  |
| Subject | Intercept | 0.046 | 0.214 |  |  |  | |  |  |  |  |
| Subject | Anger | 0.559 | 0.748 | -0.55 |  |  | |  |  |  |  |
| Subject | Fear | 0.442 | 0.665 | -0.36 | 0.92 |  | |  |  |  |  |
| Subject | Happiness | 0.364 | 0.603 | 0.37 | -0.59 | -0.58 | |  |  |  |  |
| Subject | Pleasure | 0.363 | 0.602 | 0.60 | -0.88 | -0.80 | | 0.53 |  |  |  |
| Subject | Sadness | 0.384 | 0.620 | -0.60 | 0.80 | 0.73 | | -0.73 | -0.94 |  |  |
| Subject | Stimulus Music | 0.130 | 0.360 | -0.20 | -0.02 | -0.03 | | -0.24 | -0.15 | 0.32 |  |
| Subject | Stimulus Vocals | 0.032 | 0.180 | -0.88 | 0.52 | 0.36 | | -0.68 | -0.47 | 0.59 | 0.30 |
| Residual |  | 0.890 | 0.944 |  |  |  | |  |  |  |  |

*Supplementary Table 3(a). Model estimates of the full arousal model to test the fixed and interaction effects of stimulus type and emotion category on arousal ratings: Formula = Arousal ~ Stimulus * Emotion + (1 + Emotion + Stimulus | Subject) + (1 | Item). *=<0.05. Total number of observations: 2948, number of subjects: 21, number of items: 156*

|  | Estimate | SE | *t* value | *p* | Sig |
| --- | --- | --- | --- | --- | --- |
| (Intercept) | 4.810 | 0.203 | 23.7 | <0.001 | * |
| Stimulus Vocalisations | -0.448 | 0.281 | -1.735 | 0.0846 | . |
| Stimulus Music | -0.116 | 0.263 | -0.442 | 0.6593 |  |
| Emotion Anger | 1.616 | 0.280 | 5.775 | <0.001 | * |
| Emotion Fear | 1.967 | 0.317 | 6.202 | <0.001 | * |
| Emotion Happiness | 2.053 | 0.287 | 7.152 | <0.001 | * |
| Emotion Pleasure | 0.557 | 0.326 | 1.708 | 0.0896 | . |
| Emotion Sadness | -1.742 | 0.294 | -5.917 | <0.001 | * |
| Vocalisations:Anger | 0.835 | 0.387 | 2.152 | 0.0351 | * |
| Music:Fear | 0.219 | 0.373 | 0.588 | 0.5575 |  |
| Vocalisations:Happiness | 0.241 | 0.381 | 0.633 | 0.5276 |  |
| Music:Happiness | 0.219 | 0.340 | 0.646 | 0.5191 |  |
| Vocalisations:Sadness | 2.973 | 0.381 | 7.794 | <0.001 | * |
| Music:Sadness | 1.543 | 0.340 | 4.545 | <0.001 | * |

*Supplementary Table 3(b). Random effect variance estimates for the arousal model described in Supplementary Table 3(a).*

| Group | Effect | Variance | *SD* | *Correlation* |  |  |  |  |  |  |
| --- | --- | --- | --- | --- | --- | --- | --- | --- | --- | --- |
| Item | Intercept | 0.425 | 0.652 |  |  |  |  |  |  |  |
| Subject | Intercept | 0.286 | 0.535 |  |  |  |  |  |  |  |
| Subject | Stimulus Music | 0.319 | 0.565 | -0.65 |  |  |  |  |  |  |
| Subject | Stimulus Vocals | 0.176 | 0.419 | -0.81 | 0.81 |  |  |  |  |  |
| Subject | Anger | 0.391 | 0.625 | -0.71 | 0.33 | 0.56 |  |  |  |  |
| Subject | Fear | 0.431 | 0.657 | -0.59 | 0.06 | 0.48 | 0.88 |  |  |  |
| Subject | Happiness | 0.501 | 0.708 | -0.70 | 0.29 | 0.49 | 0.94 | 0.72 |  |  |
| Subject | Pleasure | 0.537 | 0.733 | -0.39 | 0.33 | 0.03 | 0.42 | 0.14 | 0.45 |  |
| Subject | Sadness | 0.514 | 0.717 | -0.38 | 0.23 | 0.22 | 0.19 | 0.28 | 0.04 | 0.42 |
| Residual |  | 1.121 | 1.059 |  |  |  |  |  |  |  |

*Supplementary Table 4(a). Full model estimates, standard errors and p values for the speech emotion category model, model formula: erpd ~ Linear + Cubic + Quadratic * Emotion + Llinear + Quadratic + Cubic | Subject:Emotion) + Linear + Quadratic + Cubic| Subject). *p<0.05. Total number of observations: 147258, number of subjects: 21, number of subject:emotion: 84*

|  | Estimate | SE | *t* value | p | Sig |
| --- | --- | --- | --- | --- | --- |
| (Intercept) | 0.762 | 0.402 | 1.894 | 0.05817 |  |
| Linear term | -2.039 | 2.024 | -1.008 | 0.31365 |  |
| Quadratic term | -6.112 | 1.589 | -3.847 | <0.001 | * |
| Cubic term | 1.228 | 0.518 | 2.369 | 0.01783 | * |
| Anger | 0.783 | 0.243 | 3.220 | 0.00128 | * |
| Happiness | 0.662 | 0.243 | 2.727 | 0.00639 | * |
| Sadness | 0.849 | 0.243 | 3.495 | <0.001 | * |
| Quadratic:Anger | -3.797 | 1.277 | -2.973 | 0.00295 | * |
| Quadratic:Happiness | -3.111 | 1.278 | -2.434 | 0.01492 | * |
| Quadratic:Sadness | -3.309 | 1.277 | -2.590 | 0.00960 | * |

*Supplementary Table 4(b). Random effect variance estimates for the categorical speech model described in Supplementary Table 4(a).*

| Group | Effect | Variance | *SD* | *Correlation* |  |  |
| --- | --- | --- | --- | --- | --- | --- |
| Subject:Emotion | Intercept | 0.882 | 0.939 |  |  |  |
| Subject:Emotion | Linear | 31.986 | 5.656 | 0.56 |  |  |
| Subject:Emotion | Quadratic | 16.736 | 4.091 | -0.62 | -0.07 |  |
| Subject:Emotion | Cubic | 3.439 | 1.854 | -0.20 | -0.41 | 0.27 |
| Subject | Intercept | 2.711 | 1.646 |  |  |  |
| Subject | Linear | 77.700 | 8.815 | 0.60 |  |  |
| Subject | Quadratic | 35.654 | 5.971 | -0.72 | -0.01 |  |
| Subject | Cubic | 4.469 | 2.114 | 0.38 | -0.48 | -0.77 |
| Residual |  | 13.630 | 3.692 |  |  |  |

*Supplementary Table 5(a). Full model estimates, standard errors and p values for the music emotion category model: erpd ~ Linear + Quadratic + Cubic) * emotion + (Linear + Quadratic + Cubic | Subject) + (Linear + Quadratic + Cubic | Subject:Emotion). *p<0.05. Total number of observations: 173988, number of subjects: 21, number of subject:emotion: 84*

|  | Estimate | SE | *t* value | *p* | Sig |
| --- | --- | --- | --- | --- | --- |
| (Intercept) | 0.473 | 0.542 | 0.873 | 0.38247 |  |
| Linear time term | -3.744 | 2.426 | -1.543 | 0.12272 |  |
| Quadratic time term | -3.592 | 1.353 | -2.654 | 0.00795 | * |
| Cubic time term | 0.904 | 0.774 | 1.168 | 0.24297 |  |
| Emotion Fear | 1.026 | 0.294 | 3.495 | <0.001 | * |
| Emotion Happiness | 0.882 | 0.294 | 3.006 | 0.00265 | * |
| Emotion Sadness | 0.745 | 0.294 | 2.538 | 0.01115 | * |
| Linear:Fear | -3.595 | 1.866 | -1.927 | 0.05395 |  |
| Linear:Happiness | 1.429 | 1.865 | 0.766 | 0.44355 |  |
| Linear:Sadness | 5.394 | 1.866 | 2.891 | 0.00385 | * |
| Quadratic:Fear | -3.242 | 1.155 | -2.808 | 0.00499 | * |
| Quadratic:Happiness | -5.449 | 1.154 | -4.722 | <0.001 | * |
| Quadratic:Sadness | -3.191 | 1.155 | -2.762 | 0.005752 | * |
| Cubic:Fear | 3.818 | 0.806 | 4.735 | <0.001 | * |
| Cubic:Happiness | 1.195 | 0.805 | 1.484 | 0.13773 |  |
| Cubic:Sadness | -1.390 | 0.807 | -1.722 | 0.08507 |  |

*Supplementary Table 5(b). Random effect variance estimates for the categorical music model described in Supplementary Table 5(a).*

| Group | Effect | Variance | *SD* | *Correlation* |  |  |
| --- | --- | --- | --- | --- | --- | --- |
| Subject:emotion | Intercept | 0.897 | 0.947 |  |  |  |
| Subject:emotion | Linear | 35.211 | 5.934 | 0.56 |  |  |
| Subject:emotion | Quadratic | 12.671 | 3.560 | -0.69 | -0.29 |  |
| Subject:emotion | Cubic | 5.498 | 2.345 | -0.03 | -0.67 | 0.00 |
| Subject | Intercept | 5.255 | 2.292 |  |  |  |
| Subject | Linear | 87.072 | 9.331 | 0.68 |  |  |
| Subject | Quadratic | 24.462 | 4.946 | -0.74 | -0.23 |  |
| Subject | Cubic | 5.753 | 2.399 | 0.50 | -0.26 | -0.70 |
| Residual |  | 16.902 | 4.111 |  |  |  |

*Supplementary Table 6. Post hoc results for the music emotion category model for each of the time terms of the polynomial. Adjusted p-values are reported using the single-step method. *p<0.05*

|  | Estimate | SE | Z value | *p* |  |
| --- | --- | --- | --- | --- | --- |
| Fear vs. Neutral | -1.026 | 0.294 | -3.495 | 0.0104 | * |
| Fear vs. Happiness | -0.144 | 0.294 | -0.490 | 1.0000 |  |
| Fear vs. Sadness | -0.281 | 0.294 | -0.957 | 0.9914 |  |
| Neutral vs. Happiness | 0.882 | 0.294 | 3.006 | 0.0501 | . |
| Neutral vs. Sadness | 0.745 | 0.294 | 2.538 | 0.1752 |  |
| Happiness vs. Sadness | -0.137 | 0.294 | -0.468 | 1.0000 |  |
| Linear: Fear vs. Neutral | 3.595 | 1.866 | 1.927 | 0.5477 |  |
| Linear: Fear vs. Happiness | 5.025 | 1.865 | 2.694 | 0.1195 |  |
| Linear: Fear vs. Sadness | 8.989 | 1.866 | 4.817 | <0.001 | * |
| Linear: Neutral vs. Happiness | 1.429 | 1.865 | 0.766 | 0.9987 |  |
| Linear: Neutral vs. Sadness | 5.394 | 1.866 | 2.891 | 0.0698 | . |
| Linear: Happiness vs. Sadness | 3.965 | 1.865 | 2.125 | 0.4055 |  |
| Quadratic: Fear vs. Neutral | 3.242 | 1.155 | 2.808 | 0.0882 | . |
| Quadratic: Fear vs. Happiness | -2.207 | 1.154 | -1.912 | 0.5593 |  |
| Quadratic: Fear vs. Sadness | 0.052 | 1.156 | 0.45 | 1.0000 |  |
| Quadratic: Neutral vs. Happiness | -5.449 | 1.154 | -4.722 | <0.001 | * |
| Quadratic: Neutral vs. Sadness | -3.191 | 1.155 | -2.762 | 0.0997 | . |
| Quadratic: Happiness vs. Sadness | 2.258 | 1.155 | 1.956 | 0.5268 |  |
| Cubic: Fear vs. Neutral | -3.818 | 0.806 | -4.735 | <0.001 | * |
| Cubic: Fear vs. Happiness | -2.623 | 0.805 | -3.257 | 0.0230 | * |
| Cubic: Fear vs. Sadness | -5.208 | 0.807 | -6.451 | <0.001 | * |
| Cubic: Neutral vs. Happiness | 1.195 | 0.805 | 1.484 | 0.8474 |  |
| Cubic: Neutral vs. Sadness | -1.390 | 0.807 | -1.722 | 0.6986 |  |
| Cubic: Happiness vs. Sadness | -2.585 | 0.806 | -3.207 | 0.0267 | * |

*Supplementary Table 7(a). Full Model estimates and significance codes for the full vocalisations emotion category model, showing the interaction of each emotion category with each time term. Full model: erpd ~ (Linear + Quadratic) * Emotion + Cubic + (Linear+ Quadratic + Cubic | Subject:Emotion) + (Linear+ Quadratic + Cubic | Subject). *p<0.05. Total number of observations: 168804, number of subjects: 21, number of subject:emotion: 158*

|  | Estimate | SE | *t* value | *p* | Sig |
| --- | --- | --- | --- | --- | --- |
| (Intercept) | 1.109 | 0.463 | 2.393 | 0.01671 | * |
| Linear time term | 3.608 | 0.809 | 4.461 | <0.001 | * |
| Quadratic time term | -6.787 | 2.665 | -2.547 | 0.01087 | * |
| Cubic time term | -6.285 | 1.580 | -3.979 | <0.001 | * |
| Emotion Anger | 0.400 | 0.367 | 1.090 | 0.27558 |  |
| Emotion Fear | 0.056 | 0.367 | 0.153 | 0.87839 |  |
| Emotion Happiness | 1.206 | 0.367 | 3.287 | 0.00101 | * |
| Emotion Pleasure | 1.018 | 0.367 | 2.775 | 0.00552 | * |
| Emotion Sadness | 2.051 | 0.367 | 5.591 | <0.001 | * |
| Linear:Anger | 0.885 | 1.926 | 0.460 | 0.64585 |  |
| Linear:Fear | 0.598 | 1.927 | 0.310 | 0.75649 |  |
| Linear:Happiness | 1.199 | 1.928 | 0.622 | 0.53400 |  |
| Linear:Pleasure | 1.892 | 1.926 | 0.982 | 0.32608 |  |
| Linear:Sadness | 8.248 | 1.927 | 4.281 | <0.001 | * |
| Quadratic:Anger | -1.590 | 1.400 | -1.135 | 0.25633 |  |
| Quadratic:Fear | -0.172 | 1.401 | -0.122 | 0.90253 |  |
| Quadratic:Happiness | -5.823 | 1.402 | -4.153 | <0.001 | * |
| Quadratic:Pleasure | -5.369 | 1.401 | -3.833 | <0.001 | * |
| Quadratic:Sadness | -4.715 | 1.401 | -3.366 | <0.001 | * |

*Supplementary Table 7(b). Random effect variance estimates for the categorical vocalisation model described in Table 7.*

| Group | Effect | Variance | *SD* | *Correlation* |  |  |
| --- | --- | --- | --- | --- | --- | --- |
| Subject:emotion | Intercept | 1.418 | 1.191 |  |  |  |
| Subject:emotion | Linear | 45.574 | 6.751 | 0.36 |  |  |
| Subject:emotion | Quadratic | 19.129 | 4.374 | -0.46 | 0.19 |  |
| Subject:emotion | Cubic | 8.062 | 2.839 | 0.12 | -0.48 | -0.17 |
| Subject | Intercept | 3.090 | 1.758 |  |  |  |
| Subject | Linear | 108.762 | 10.429 | 0.48 |  |  |
| Subject | Quadratic | 31.733 | 5.633 | -0.70 | 0.06 |  |
| Subject | Cubic | 12.076 | 3.475 | 0.35 | -0.55 | -0.58 |
| Residual |  | 15.700 | 3.962 |  |  |  |

*Supplementary Table 8 Post hoc estimates for each of the pairwise comparisons of the full vocalisations emotion category model. P values are adjusted according to the single step method. *p<0.05*

|  | Estimate | | SE | Z value | *p* | Sig |
| --- | --- | --- | --- | --- | --- | --- |
| Pleasure vs. Neutral | | -1.018 | 0.367 | -2.775 | 0.1652 |  |
| Pleasure vs. Anger | | -0.618 | 0.367 | -1.685 | 0.8762 |  |
| Pleasure vs. Fear | | -0.962 | 0.367 | -2.622 | 0.2375 |  |
| Pleasure vs. Happiness | | 0.188 | 0.367 | 0.513 | 1.0000 |  |
| Pleasure vs. Sadness | | 1.033 | 0.367 | 2.816 | 0.1475 |  |
| Neutral vs. Anger | | 0.400 | 0.367 | 1.090 | 0.9970 |  |
| Neutral vs. Fear | | 0.056 | 0.367 | 0.153 | 1.0000 |  |
| Neutral vs. Happiness | | 1.206 | 0.367 | 3.287 | 0.0382 | * |
| Neutral vs. Sadness | | 2.051 | 0.367 | 5.591 | <0.001 | * |
| Anger vs. Fear | | -0.344 | 0.367 | -0.937 | 0.9994 |  |
| Anger vs. Happiness | | 0.806 | 0.367 | 2.197 | 0.5262 |  |
| Anger vs. Sadness | | 1.651 | 0.367 | 4.501 | <0.001 | * |
| Fear vs. Happiness | | 1.150 | 0.367 | 3.134 | 0.0602 | . |
| Fear vs. Sadness | | 1.995 | 0.367 | 5.438 | <0.001 | * |
| Happiness vs. Sadness | | 0.845 | 0.367 | 2.303 | 0.4452 |  |
| Linear: Pleasure vs. Neutral | | -1.892 | 1.926 | -0.982 | 0.9990 |  |
| Linear: Pleasure vs. Anger | | -1.007 | 1.927 | -0.523 | 1.0000 |  |
| Linear: Pleasure vs. Fear | | -1.294 | 1.927 | -0.672 | 1.0000 |  |
| Linear: Pleasure vs. Happiness | | -0.693 | 1.929 | -0.359 | 1.0000 |  |
| Linear: Pleasure vs. Sadness | | 6.356 | 1.927 | 3.299 | 0.0362 | * |
| Linear: Neutral vs. Anger | | 0.885 | 1.926 | 0.460 | 1.0000 |  |
| Linear: Neutral vs. Fear | | 0.597 | 1.927 | 0.310 | 1.0000 |  |
| Linear: Neutral vs. Happiness | | 1.199 | 1.928 | 0.622 | 1.0000 |  |
| Linear: Neutral vs. Sadness | | 8.248 | 1.927 | 4.281 | <0.001 | * |
| Linear: Anger vs. Fear | | -0.287 | 1.927 | -0.149 | 1.0000 |  |
| Linear: Anger vs. Happiness | | 0.314 | 1.929 | 0.163 | 1.0000 |  |
| Linear: Anger vs. Sadness | | 7.363 | 1.927 | 3.821 | <0.01 | * |
| Linear: Fear vs. Happiness | | 0.602 | 1.929 | 0.312 | 1.0000 |  |
| Linear: Fear vs. Sadness | | 7.650 | 1.927 | 3.969 | <0.01 | * |
| Linear: Happiness vs. Sadness | | 7.049 | 1.929 | 3.654 | 0.0102 | * |
| Quadratic: Pleasure vs. Neutral | | 5.369 | 1.401 | 3.833 | <0.01 | * |
| Quadratic: Pleasure vs. Anger | | 3.779 | 1.401 | 2.698 | 0.1993 |  |
| Quadratic: Pleasure vs. Fear | | 5.197 | 1.401 | 3.709 | <0.01 | * |
| Quadratic: Pleasure vs. Happiness | | -0.455 | 1.402 | -0.324 | 1.0000 |  |
| Quadratic: Pleasure vs. Sadness | | 0.654 | 1.401 | 0.467 | 1.0000 |  |
| Quadratic: Neutral vs. Anger | | -1.590 | 1.400 | -1.135 | 0.9955 |  |
| Quadratic: Neutral vs. Fear | | -0.172 | 1.401 | -0.122 | 1.0000 |  |
| Quadratic: Neutral vs. Happiness | | -5.823 | 1.402 | -4.153 | <0.01 | * |
| Quadratic: Neutral vs. Sadness | | -4.715 | 1.401 | -3.366 | 0.0289 | * |
| Quadratic: Anger vs. Fear | | 1.418 | 1.401 | 1.012 | 0.9986 |  |
| Quadratic: Anger vs. Happiness | | -4.234 | 1.402 | -3.019 | 0.0845 | . |
| Quadratic: Anger vs. Sadness | | -3.125 | 1.401 | -2.231 | 0.4987 |  |
| Quadratic: Fear vs. Happiness | | -5.652 | 1.403 | -4.029 | <0.01 | * |
| Quadratic: Fear vs. Sadness | | -4.543 | 1.401 | -3.242 | 0.0433 | * |
| Quadratic: Happiness vs. Sadness | | 1.108 | 1.403 | 0.790 | 0.9999 |  |

*Supplementary Table 9 Random effect variance estimates for the valence and arousal stimuli model described in Table 7 of the manuscript.*

| Group | Effect | Variance | *SD* | *Correlation* |  |  |
| --- | --- | --- | --- | --- | --- | --- |
| Item | Intercept | 1.191 | 1.091 |  |  |  |
| Item | Linear | 40.609 | 6.373 | 0.46 |  |  |
| Item | Quadratic | 17.353 | 4.166 | -0.69 | -0.12 |  |
| Item | Cubic | 8.385 | 2.896 | 0.03 | -0.68 | -0.02 |
| Subject | Intercept | 3.266 | 1.807 |  |  |  |
| Subject | Linear | 83.327 | 9.128 | 0.54 |  |  |
| Subject | Quadratic | 30.887 | 5.558 | -0.71 | 0 |  |
| Subject | Cubic | 7.468 | 2.733 | 0.37 | -0.50 | -0.63 |
| Residual |  | 16.049 | 4.006 |  |  |  |

**Supplementary Figure Captions**

Supplementary Figure 1 Confusion matrix of the behavioural recognition scores of the speech stimuli. The y-axis presents the emotion category label given by the participants, and the x-axis shows the emotion expressed by the actors.


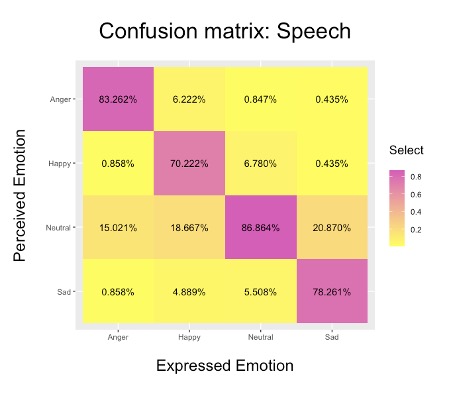


Supplementary Figure 2 Confusion matrix of the behavioural recognition scores of the music stimuli. The y-axis presents the emotion category label given by the participants, and the x-axis shows the emotion expressed by the actors.


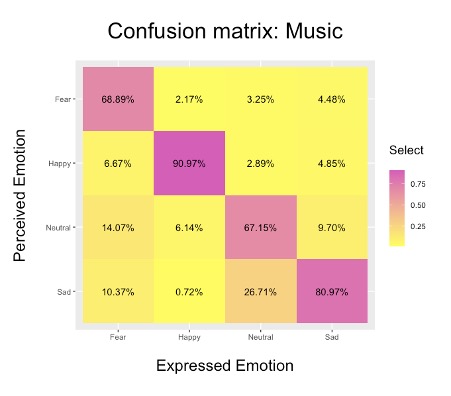


Supplementary Figure 3 Confusion matrix of the behavioural recognition scores of the vocalisation stimuli. The y-axis presents the emotion category label given by the participants, and the x-axis shows the emotion expressed by the actors.

**
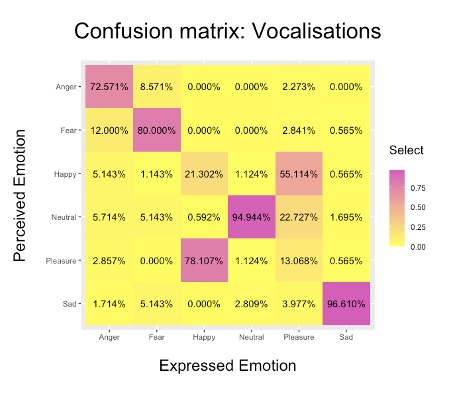
**
